# Description of a neural sheath tumor of the trigeminal nerve: immunohistochemical and electron microscopy study

**DOI:** 10.1590/S1516-31802006000600006

**Published:** 2006-11-01

**Authors:** Bijan Khademi, Seied Mohammad Owji, Khadije Jamshidi Khosh, Mohammad Mohammadianpanah, Behrooz Gandomi

**Keywords:** Nerve sheath neoplasms, Paranasal sinuses, Trigeminal nerve, Radiotherapy, Chemotherapy, Neoplasias da bainha neural, Seios paranasais, Nervo trigêmeo, Radioterapia, Quimioterapia

## Abstract

**CONTEXT:**

Malignant neural sheath tumors of the trigeminal nerve affecting the nasal cavity and the paranasal sinuses are extremely rare. With conventional optical microscopy, their identification is difficult, and it is necessary to confirm them by means of electron microscopy and immunohistochemical techniques.

**CASE REPORT:**

The patient was a 41-year-old woman with a ten-month progressive history of pain followed by painful edema in the left facial region, and with symptoms of bleeding, secretion and nasal obstruction. Studies with imaging methods suggested the presence of an expansive process in the left nasal and paranasal cavities. In the biopsy, the histopathological findings from optical microscopy were suggestive of a tumor of neural origin in the trigeminal nerve. Immunohistochemical and electron microscopy studies confirmed that it was a malignant tumor of the neural sheath of the trigeminal nerve. We describe the clinical, radiological, and histological features of this tumor and review the literature.

## RESUMO

**CONTEXTO:**

O tumor maligno da bainha neural do trigêmeo que acomete a cavidade nasal e os seiosparanasais é extremamente raro. À microscopia óptica habitual sua identificação é difícil, sendo necessária a confirmação através de microscopia eletrônica e de técnicas imunoistoquímicas.

**RELATO DE CASO:**

A paciente era uma mulher de 41 anos com história de 10 meses de progressiva dor seguida de edema álgico em região facial à esquerda, e de sintomas de sangramento, secreção e obstrução nasal. Estudos com métodos de imagem sugeriam a presença de processo expansivo em cavidades nasaise paranasais à esquerda. À biopsia, os achados histopatológicos à microscopia óptica foram sugestivos de tumor de origem neural no trigêmeo. Estudos imunoistoquímico e por microscopia eletrônica confirmaram se tratar de neoplasia maligna da bainha neural do trigêmeo. Descrevemos as características clínicas, radiológicas e histológicas deste tumor, e revisamos a literatura.

## INTRODUCTION

Sarcomas of the head and neck region are a rare and diverse group of neoplasms, accounting for less than 1% of all neoplasms that occur in this area.^1^ Primary neurogenic tumor of the nose and paranasal sinuses account for only 4% of all neural tumors of the head and neck region.^2^ Malignant peripheral nerve sheath tumor (MPNST) of the trigeminal nerve affecting the sinonasal region is rare, and a search in PubMed revealed only 20 cases reported so far. The extremities, trunk, chest and retroperitoneum are the most common primary sites for this aggressive neoplasm.^3,4^

Here, we describe a case with malignant peripheral nerve sheath tumor (MPNST) of the maxillary sinus, studied by means of electron microscopy.

## CASE REPORT

A 41-year-old woman presented with a 10-month history of progressive toothache that was followed by painful swelling of the left cheek, epistaxis, nasal obstruction and discharge. On examination, a tender swelling of the left cheek was found. Intraoral examination showed an ulcerative, hard mass in the left upper alveolar ridge. There was some left orbital edema.

**Figure 1:**
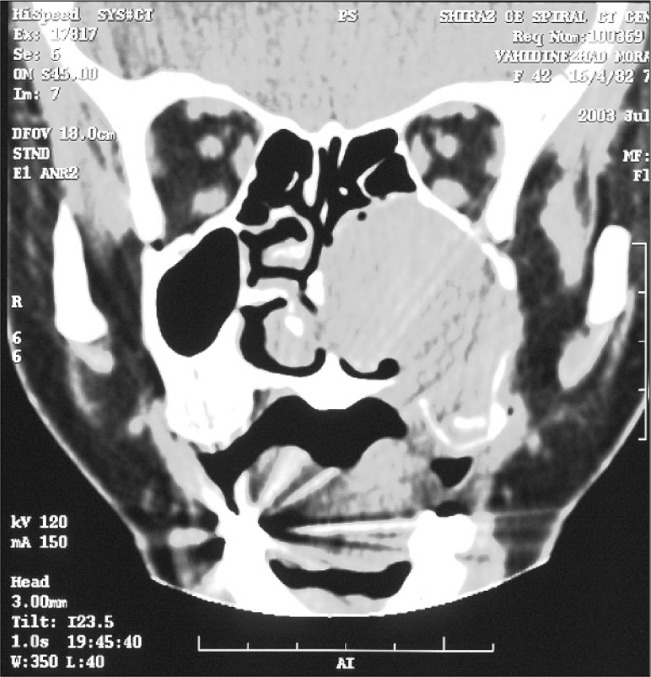
Coronal computed tomography scan from the nose and paranasal sinuses, showing a large mass in the left maxillary sinus with extension to the nasal cavity, in a 41-year-old woman.

**Figure 2:**
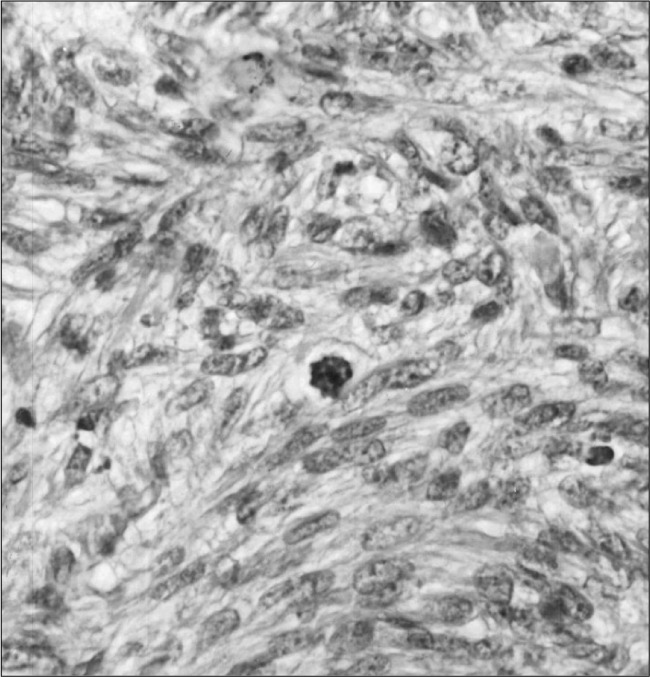
Interlacing fascicles of tightly packed ovoid to spindle cells, bearing hyperchromatic nuclei and indistinct cytoplasm with high mitotic rate in a biopsy from the maxillary sinus (hematoxylineosin, 400 X).

**Figure 3:**
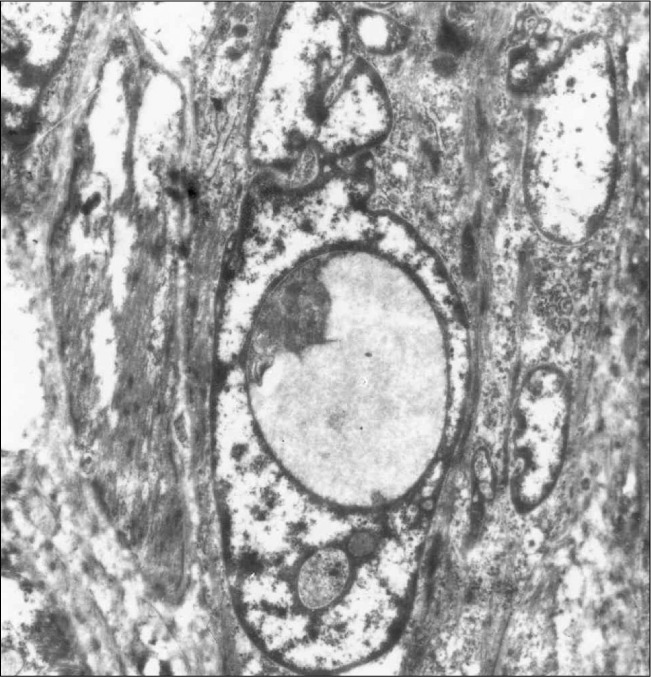
Electron microscopy of tumor cells in a peripheral nerve sheath tumor, demonstrating elongated ovoid cells with thin cytoplasmic processes within a collagen matrix (uranium acetate and lead citrate, 10,000 X).

**Figure 4:**
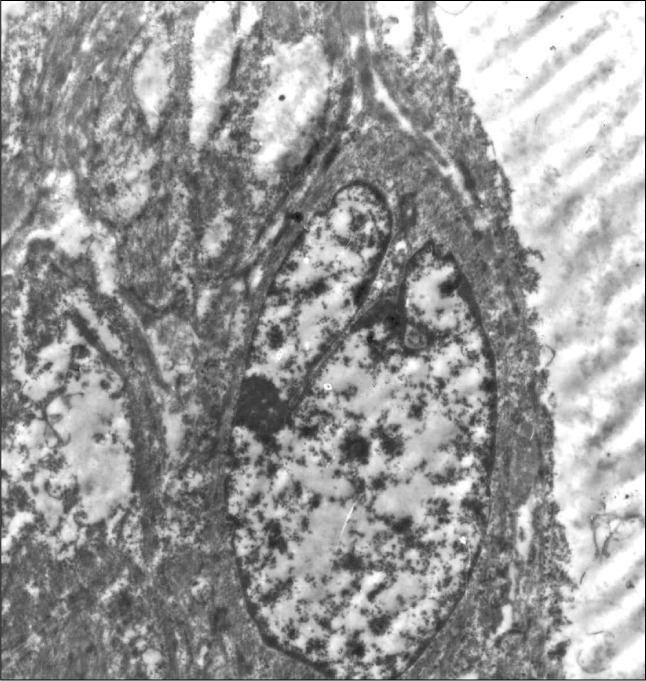
Presence of basal lamina around the cells, showing tonofilaments in the cytoplasm and branching of the nucleus of a peripheral nerve sheath tumor (uranium acetate and lead citrate, 10,000 X).

**Figure 5:**
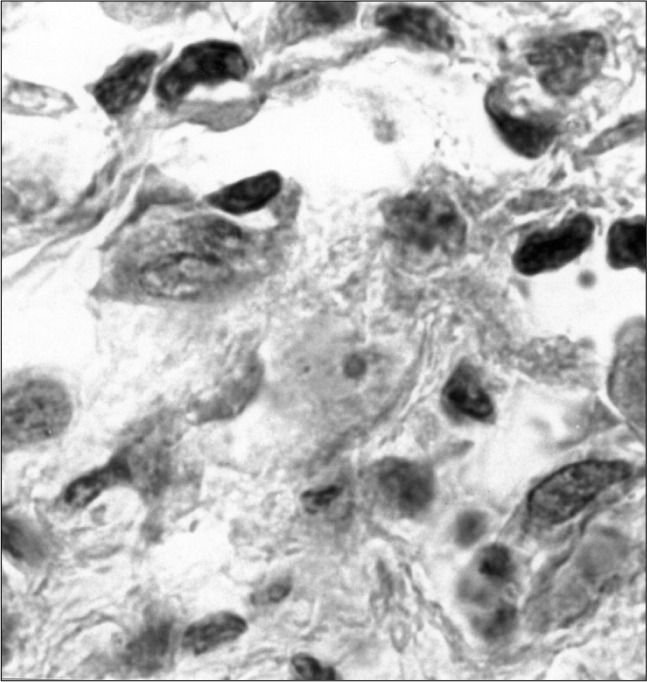
Tumor cells with positive reactivity for S100 (1,000 X) in immunochemistry of a peripheral nerve sheath tumor.

**Figure 6:**
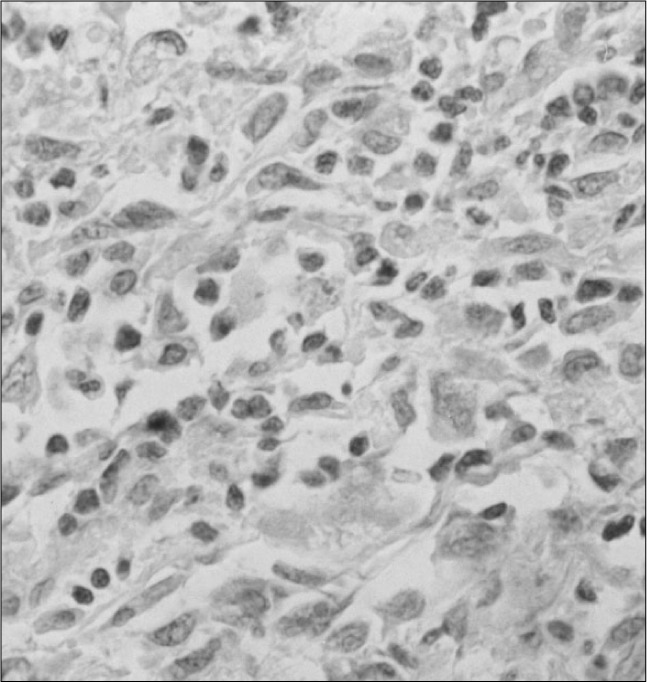
Tumor cells with positive reacti vity for vimentin (220 X) in immunochemistry of a peripheral nerve sheath tumor.

Computed tomography (CT) scans showed a space-occupying lesion arising from the left maxillary sinus, causing expansion of the left maxillary sinus. This mass extended medially to the nasal cavity, superiorly to the floor of the left orbit, and inferiorly to the left upper alveolar ridge (Figure 1). A biopsy from the maxillary sinus revealed spindle cell sarcoma, suggestive of peripheral nerve sheath tumor (Figure 2). Electron microscopy revealed elongated ovoid tumor cells with thin cytoplasmic processes within a collagen matrix (Figure 3). The presence of basal lamina around the cells, tonofilaments in the cytoplasm and branching of the nucleus were diagnostic of MPNST (Figure 4). Immunohistochemistry studies demonstrated moderate focal positivity for S100 (Figure 5) and vimentin (Figure 6), thus confirming the diagnosis of MPNST of the trigeminal nerve.

The patient underwent left maxillectomy with curative intent. However, the disease was too extensive: gross residual tumor was left behind and complete tumor clearance could not be obtained surgically. Because of the persistent gross residual disease, the patient received a combination of chemotherapy and radiation therapy. The chemotherapy course consisted of 4 cycles of cyclophosphamide (1000 mg/m^2^), vincristine sulfate (1.4 mg/m^2^) and doxorubicin (50 mg/m^2^) once every three weeks. By the end of the fourth cycle of treatment, there had still not been any significant clinical response to the chemotherapy. Therefore, the patient was treated with a radical radiation therapy using an anterior and lateral paired wedged field technique, and a dose of 70 Gy was delivered in 35 daily fractions. Complete response was achieved by the end of the radiation therapy. During 26 months of follow-up after terminating the radiotherapy, there was no evidence of locoregional and distant failure.

## DISCUSSION

Sarcomas account for 24% of all nonsquamous cell malignant tumors of the sinonasal region.^5^ MPNST, also known as malignant schwannoma, malignant neurilemoma, neurogenic sarcoma, and neurofibrosarcoma, is a rare neoplasm that originates in Schwann's cells of the nerve sheath.^3^ Most nerve sheath tumors of the nose and paranasal sinuses arise from the ophthalmic and maxillary branches of the trigeminal nerve and its terminal branches, although it is often difficult to identify exactly which nerve is involved. The most common sites for these tumors are the ethmoid and maxillary sinuses. Other sites are the nasal cavity and the sphenoid sinus. The frontal sinus is the least common site.^6,7^ MPNSTs can arise de novo or as a result of the malignant transformation of a neurofibroma (Von Recklinghausen's disease). These tumors occur in all age groups and in both sexes, but the peak incidence is between the second and fourth decades of life. The tumor has no predilection for sex and race.^8^

The clinical presentation differs according to the site involved, but the most common symptoms are unilateral nasal obstruction, epistaxis, pain in the facial region and swelling of the facial and orbital region. The other complaints presented include mucopurulent rhinorrhea, hyposmia and headache.^9^ It must be remembered that nasal obstruction has a wide variety of clinical differential diagnoses and, in cases of spontaneous extrusion of the teeth in the maxillary alveolar arch, the pathological lesion in the maxillary sinus must be investigated.

### Histopathological characteristics

Histologically, MPNST has no classical appearance and it is often confounded with other tumors such as fibrosarcoma, rhabdomyosarcoma, esthesioneuroblastoma, poorly differentiated lymphoma and amelanotic melanoma.^10,11^

It is difficult to use optical microscopy alone to differentiate MPNST from other spindle cell sarcomas, especially if its origin from a nerve trunk cannot be identified. The interpretation is based on optical microscopy sampling as well as the clinical and gross anatomical contexts of each case, while immunohistochemistry findings of S100 and vimentin are diagnostic. Electron microscopic investigation may yield important clues for final diagnosis of MPNST. Intracytoplasmic tonofilaments and collections of dense-core granules^12,13^ have been found in most cases of MPNST. The latter was not seen in the present case. Long-spaced collagen has been seen in other benign or malignant neurogenic tumors. The overall ultrastructural features that differentiate Schwann cell origin from other spindle cells are characterized by long, slender cytoplasmic processes enveloping each other and other cells. These processes are outlined by a discrete basal lamina and also tonofilaments and dense core granules in the cytoplasm of tumor cells.^12,13^ On the other hand, the electron microscopic findings from other tumors that are diagnosed differentially from MPNST are histologically distinct and well defined.

### Clinical course and treatment

Local recurrence is the major cause of treatment failure and poor overall survival in these patients.^5^ Regional metastasis is infrequent. Sarcomas involving the sinonasal region may have an indolent course for long-lasting periods, and may present with only nasal obstruction or drainage in the absence of orbital involvement. Combined therapy is the cornerstone of treatment for most soft tissue sarcomas of the head and neck region.^14^ Wide surgical resection is the primary treatment for neurogenic sarcomas. Because of the tendency for neurogenic sarcomas to recur locally, postoperative radiation therapy is recommended by many physicians.^15^ Radiation therapy significantly improves survival for patients with T4 lesions.^14^

Doxorubicin-based adjuvant chemotherapy appears to improve disease-free survival and probably improves overall survival in adults with localized resectable head and neck soft tissue sarcoma. However, the role of adjuvant chemotherapy in neurogenic sarcomas remains poorly defined.^15,16^

## CONCLUSION

The paranasal sinuses are not a common location for MPNST, but if the clinical and radiological investigations and the gross and histological findings are consistent with MPNST, electron microscopy is suggested for confirmation of the final diagnosis.
